# Genetic and Dietary Factors Influencing the Progression of Nuclear Cataract

**DOI:** 10.1016/j.ophtha.2016.01.036

**Published:** 2016-06

**Authors:** Ekaterina Yonova-Doing, Zoe A. Forkin, Pirro G. Hysi, Katie M. Williams, Tim D. Spector, Clare E. Gilbert, Christopher J. Hammond

**Affiliations:** 1 Department of Twin Research and Genetic Epidemiology, Kings College London, London, United Kingdom; 2 Department of Ophthalmology, Kings College London, London, United Kingdom; 3 University of Warwick Medical School, Coventry, United Kingdom; 4 London School of Hygiene and Tropical Medicine, London, United Kingdom

**Keywords:** CI, confidence interval, DZ, dizygotic, FFQ, food frequency questionnaire, HATS, Healthy Ageing in Twins, LOCS, Lens Opacity Classification System, MZ, monozygotic, NDS, nuclear dip score, RRR, relative risk ratio, SD, standard deviation

## Abstract

**Purpose:**

To determine the heritability of nuclear cataract progression and to explore prospectively the effect of dietary micronutrients on the progression of nuclear cataract.

**Design:**

Prospective cohort study.

**Participants:**

Cross-sectional nuclear cataract and dietary measurements were available for 2054 white female twins from the TwinsUK cohort. Follow-up cataract measurements were available for 324 of the twins (151 monozygotic and 173 dizygotic twins).

**Methods:**

Nuclear cataract was measured using a quantitative measure of nuclear density obtained from digital Scheimpflug images. Dietary data were available from EPIC food frequency questionnaires. Heritability was modeled using maximum likelihood structural equation twin modeling. Association between nuclear cataract change and micronutrients was investigated using linear and multinomial regression analysis. The mean interval between baseline and follow-up examination was 9.4 years.

**Main Outcome Measures:**

Nuclear cataract progression.

**Results:**

The best-fitting model estimated that the heritability of nuclear cataract progression was 35% (95% confidence interval [CI], 13–54), and individual environmental factors explained the remaining 65% (95% CI, 46–87) of variance. Dietary vitamin C was protective against both nuclear cataract at baseline and nuclear cataract progression (β = −0.0002, *P* = 0.01 and β = −0.001, *P* = 0.03, respectively), whereas manganese and intake of micronutrient supplements were protective against nuclear cataract at baseline only (β = −0.009, *P* = 0.03 and β = −0.03, *P* = 0.01, respectively).

**Conclusions:**

Genetic factors explained 35% of the variation in progression of nuclear cataract over a 10-year period. Environmental factors accounted for the remaining variance, and in particular, dietary vitamin C protected against cataract progression assessed approximately 10 years after baseline.

Age-related cataract is the leading cause of blindness in the world, affecting approximately 20 million people, particularly in sub-Saharan Africa.^1^ Its prevalence increases from 2.9% in the 43- to 54-year age group to 40% in those older than 75 years of age.^2^ As the world's population ages, cataract will remain a serious healthcare and socioeconomic burden, in terms of both healthcare provision and blindness in less-developed countries.

Nuclear cataract is the most common form of age-related cataract.^2^ Apart from age, other factors associated with nuclear cataract are smoking, oxidative stress, and dietary antioxidant intake.^3^, ^4^, ^5^ However, studies of the effect of dietary vitamin C intake,^6^, ^7^, ^8^, ^9^, ^10^, ^11^ serum vitamin C concentrations,^6^, ^9^, ^11^, ^12^, ^13^ and vitamin C supplementation^6^, ^10^, ^14^ on nuclear cataract formation often have given conflicting results. Case-control studies^7^, ^11^, ^12^, ^14^ and some cohort studies^6^, ^9^, ^10^ have found protective effects. Other prospective cohort studies have found no effect overall^8^, ^13^, ^15^ or protective effects only in subgroups.^8^, ^15^ Similarly to vitamin C, dietary^6^, ^16^ and supplemental^14^, ^17^ vitamin E intake and vitamin E blood concentrations^6^, ^13^ have been shown to be related inversely with nuclear cataract. Randomized clinical trials of vitamins C and E supplementation alone or in combination with other vitamins failed to find an effect.^18^, ^19^ Vitamin A has been associated with a reduced risk of nuclear cataract,^9^, ^20^, ^21^ as have lutein and zeaxanthin.^22^, ^23^, ^24^ The studies exploring dietary nutrients and cataract progression have findings similar to those looking at prevalent cataract, with cohort studies finding a protective effect.^16^, ^25^ However, supplement trials largely have failed to find an effect.^18^, ^26^, ^27^

As opposed to vitamins and micronutrients,^28^ the role of minerals in cataract formation in general and in nuclear cataract in particular is poorly studied. Together with epidemiologic factors, genetic factors also play a role in cataract formation. We previously reported that genetic factors explain 48% of cross-sectional variance in age-related nuclear cataract.^29^ In a recent genome-wide meta-analysis, variants in 2 genes, *CRYAA* and *KCNAB1*, were found to be associated with nuclear cataract in Asian populations,^30^ but no findings are available for populations of European origin. In comparison with epidemiologic factors, little is known about genetic susceptibility factors in age-related cataract.

Factors that lead to the development of a phenotype may be different from factors underlying change, such as progression of lens opacity. Therefore, we set out to establish the relative importance of genes on progression of nuclear cataract using a classic twin model with a highly quantitative measure of nuclear cataract. We also examined how intake of micronutrients and supplements associated with nuclear cataract at baseline affects nuclear cataract progression over a decade.

## Methods

### Subjects

**Figure 1:**
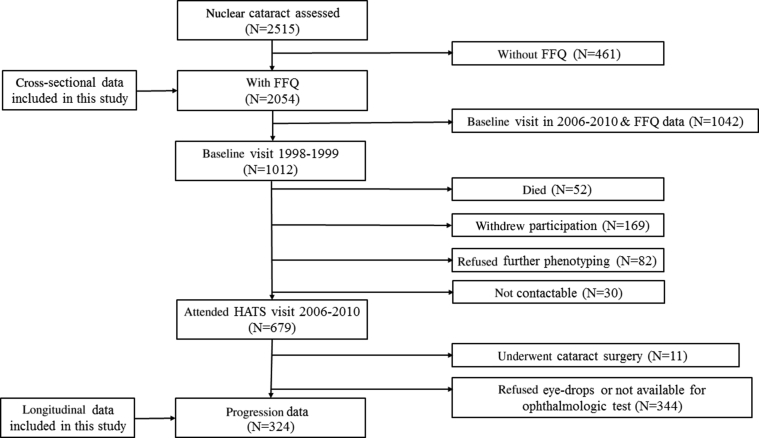
Consort diagram of the study showing the number of individuals who participated in the different parts of the study and reasons for no participation at follow-up. FFQ = food frequency questionnaire; HATS = Healthy Ageing in Twins.

Nuclear cataract data at baseline were available for 2515 white female twins (mean age, 62.3 years; range, 50.1–83.1 years) from the TwinsUK cohort, 2054 of whom had also completed a food frequency questionnaire (FFQ) around the time of their eye examination. The median time interval between an eye test taking place and a FFQ completion was 2 years. The 461 twins with cataract data but without FFQ data were 2.5 years younger on average and less affected by cataract. Cataract progression data were collected from 324 twins (151 monozygotic [MZ] and 173 dizygotic [DZ]) with a mean age at follow-up of 69.8±5.4 years (range, 58.3–83.6 years) as part of the Healthy Ageing in Twins (HATS) study between 2006 and 2010.^31^ Individuals included in the follow-up were all part of our original cataract heritability study of 1012 twin participants assessed in 1998 and 1999.^29^ The mean time between baseline and second visits was 9.4 years (range, 7–12 years). The smaller number of individuals with follow-up data is mainly due to the fact that the HATS study (in which the follow-up data were collected) was not designed specifically as a cataract follow-up study and had different selection criteria: Participants were aged more than 40 years and had to have previously attended clinical phenotyping irrespective of whether they had an eye examination or not (N = 4610) (Fig 1). The TwinsUK study started in 1992, but eye measures were performed only on subjects more than 50 years of age in 1998–1999, and subsequently from 2006. That meant that individuals (aged ≥50 years) who attended the HATS visit and who did not have eye examinations in 1989–1999 had their baseline cataract assessment during HATS (2006–2011; N = 1523). Reasons for having only longitudinal data for 324 of the original 1012 twins included death (N = 52), withdrawal of participation from the TwinsUK registry (N = 169), noncontactable (N = 30), refusal of further phenotyping (N = 82), cataract surgery (N = 11), and refusal of dilating drops or unavailability of ophthalmic testing at the HATS visit (N = 344).

Both the baseline study and the HATS study received local research ethics approval and were conducted according to the tenets of the Declaration of Helsinki. All the participants gave written informed consent.

### Phenotyping

#### Nuclear Cataract Scores

Digital black and white lens photographs were taken using a Scheimpflug camera (Case 2000; Marcher Enterprises Ltd., Worcester, UK), and the same camera was used at both baseline and follow-up. Nuclear cataract was measured quantitatively by calculating the pixel density in the center of the lens nucleus, also known as the central nuclear dip score (NDS).^29^ This score measures the amount of white scatter (opalescence), and more opacification results in higher pixel density. Because NDS uses black-and-white images, it does not assess the brunescence of the lens. Nuclear cataract progression was measured as the difference in measurements between the visits: ΔNDS = NDS at follow-up − NDS at baseline. Both NDS and ΔNDS were not normally distributed and therefore were transformed using natural logarithm before the analysis.

#### Nutrient Intake

Intake of micronutrients (vitamins and minerals) and supplements was estimated using the EPIC FFQ, which was self-administered at the baseline visit. This questionnaire explored the average frequency of intake of 131 foods and supplements over a 1-year period.^32^, ^33^ Nutrient intake was calculated using an established nutrient database, and the dietary variables were adjusted for calorie intake, yielding an energy-adjusted mg/μg of each nutrient per person per day.^32^, ^34^, ^35^ We considered the following micronutrients in the analysis: sodium, potassium, calcium, magnesium, phosphorus, iron, copper, zinc, chloride, manganese, iodine, retinol, carotene, vitamin D, vitamin E, thiamine, riboflavin, niacin, tryptophan, vitamin B6, vitamin B12, folate, pantothenate, biotin, and vitamin C.

Data on supplement intake were available for 33 different supplements. However, the percentage of individuals taking any single supplement was 10% or less. Supplements were grouped as follows: *any supplements*; *micronutrient supplements* (vitamins and minerals in any combination); *micronutrient supplements excluding multivitamins* (e.g., vitamin C only, vitamin D only, iron only, ACD complex); *minerals only* (e.g., iron only, calcium only); and *other supplements* (e.g., aloe vera, Echinacea, Ginkgo, omega-3). Each supplement group was coded as a binary variable, with “yes” indicating that they took 1 or more of the supplements in a specific group.

### Statistical Analysis

#### Modeling of Heritability

Heritability was analyzed in 310 twins (155 pairs: 72 MZ and 83 DZ) because data were missing on 14 co-twins. Zygosity was determined by a standardized questionnaire and confirmed using genome-wide single nucleotide polymorphism genotyping data or DNA short tandem repeat fingerprinting.

Twin studies are able to estimate the heritability of a trait (the amount of variance explained by genetic factors) using maximum likelihood structural equation modeling. The variance of the trait and the covariance within twin pairs are used to estimate additive genetic effects (A), shared/family environmental effects (C), and individual environmental effects (E). We implemented the modeling in the OpenMx package (http://openmx.psyc.virginia.edu). The goodness of fit of the full ACE model and submodels were compared with the observed data and the best fitting model was selected.

#### Nutrient Factor Analysis

Comparisons of means and proportions for all variables between individuals with or without follow-up data, or between MZ and DZ twins per group in terms of age, nuclear cataract scores, and nutrient and supplement intake were performed using 2-sample, 2-tailed *t* tests or *z* tests, assuming equal variance.

Association was assessed using linear regression analyses. Univariable linear regression was first carried out where each factor or supplement group was individually regressed against NDS at baseline. All nutrients or supplement groups showing significant univariable association (*P* < 0.05) were then included in a multivariable linear regression model; independent variables were identified using a stepwise backward procedure with threshold for removal set at 0.05. Factors showing significant (*P* < 0.05) association in the multivariable model were tested for association with progression. We used linear models to establish the relationship between NDS (continuous variable) and nutrients, but because NDS had to be normalized, giving a clinical interpretation of the betas becomes more difficult. Therefore, in addition to the linear models, we calculated risk reduction by calculating relative risk ratios (RRRs) using multinomial regression. In this case, NDS, ΔNDS, and the associated nutrients were divided into tertiles, and the first tertile was set as reference while supplement intake per supplement group was kept binary. In all cases, models were adjusted for family structure and age at the first visit only (baseline analysis) or for both age at baseline and Δage (age at follow-up − age at baseline). All analyses were carried using the STATA10 statistical package (StataCorp LP, College Station, TX; www.stata.com).

## Results

**Figure 2:**
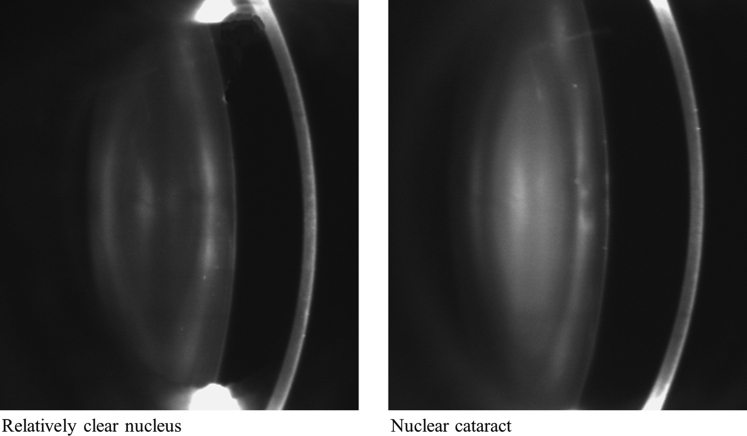
Black-and-white Scheimpflug lens images of a healthy lens (*left*) and a lens with nuclear cataract (*right*). The center of the lens (lens nucleus) on the *right* is much whiter than the one on the *left*.

Cross-sectional data were available for 2054 white female twins (827 MZ and 916 DZ), 324 (151 MZ and 173 DZ) of whom also had nuclear cataract measured at follow-up. Baseline characteristics and nutrient and supplement intake are shown in Table 1, and an example of a lens image is available in Figure 2. The twins with follow-up data were on average 1.1 years younger at baseline (60.4 vs. 61.5 years) and, given their younger age, had less cataract (mean NDS scores of 55.3 and 60.4, respectively) compared with those with only cross-sectional data. In both cases, these differences were not statistically significant (*P* > 0.05). The MZ and DZ twins with follow-up data were similar in terms of age and NDS scores (*P* > 0.05). The MZ and DZ twins with cross-sectional data only were similar in terms of age, but the MZ twins had a slightly higher NDS score (61.6 vs. 59.3; *P* = 0.02).

There were also no statistically significant differences between groups in terms of micronutrient intake except for iron (*P* = 0.02), thiamine (*P* = 0.04), and biotin (*P* = 0.01). The twins with follow-up data had slightly lower iron and thiamine intake (mean of 12.6 and 1.7 mg, respectively) and slightly higher biotin intake (mean of 49.7 mg) compared with individuals without follow-up data. There were also no significant differences in supplement intake between the 2 groups (*P* > 0.05). There were no statistically significant differences between MZ and DZ twins in terms of nutrient or supplement intake (*P* > 0.05).

**Figure 3:**
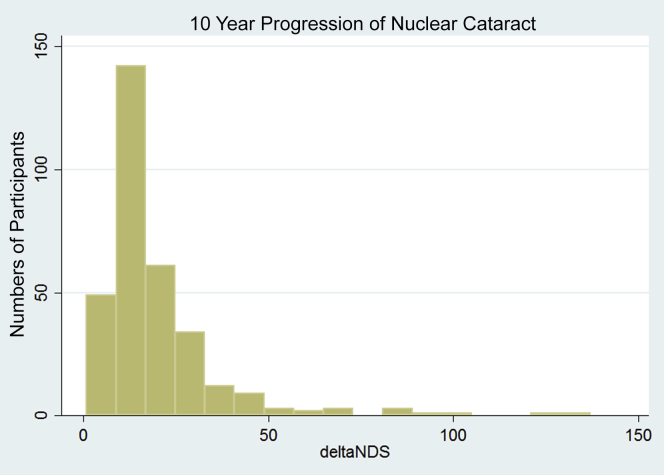
Progression of nuclear cataract between the 2 visit dates. Graphical representation of the progression of nuclear cataract (deltaNDS) between the 2 visits. The y-axes show frequency of deltaNDS per bins with width of 6.25 points. deltaNDS = NDS at follow-up − NDS at baseline; NDS = nuclear dip score.

As expected, nuclear cataract scores progressed in all participants (Fig 3). The mean baseline central NDS was 55±11 (range, 32–99) with the score increasing by an average of 19.9±16.9 (range, 1–137) over the period of follow-up. The heritability analysis, conducted on 155 twin pairs (72 MZ and 83 DZ pairs), showed that the best-fitting model was one explained by additive genetic factors and the unique (individual) environment, with no significant effect of a common environment or nonadditive genetic factors. Calculations estimated the heritability to be 0.35, meaning that genetic factors explained 35% of variance (95% confidence interval [CI], 13–54) in progression of nuclear cataract, with individual environmental factors accounting for the remaining 65% (95% CI, 46–87).

To test associations between micronutrient intake and cataract progression, we used univariable regression (Table 2) followed by stepwise regression in 2054 female twins who had baseline data on nutrient intake. Seven micronutrients showed a significant association (*P* < 0.05) with NDS and were used in multivariable analysis: potassium, magnesium, manganese, phosphorus, vitamins C and E, and folate. After stepwise multivariable regression, 2 factors remained significantly associated with NDS at baseline: vitamin C (β = −0.0002; standard deviation [SD] = 6.3E-05; *P* = 0.01) and manganese (β = −0.009; SD = 0.04; *P* = 0.03). From these 2 nutrients, only vitamin C showed association with cataract progression (β = −0.001; SD = 0.001; *P* = 0.03). A sensitivity analysis excluding subjects with greatest progression (>100 units of change) did not alter the result. Comparing people in the highest and lowest tertiles of vitamin C intake was associated with a 19% risk reduction at baseline (RRR, 0.81; 95% CI, 0.68–0.96) and a 33% risk reduction of cataract progression (RRR, 0.66; 95% CI, 0.47–0.91) (Table 3). Manganese intake was associated with 20% risk reduction (RRR, 0.80; 95% CI, 0.67–0.95) at baseline (Table 3).

Two supplement groups, *micronutrient supplements* and *minerals only*, showed a significant association with NDS (*P* < 0.05) (Table 2), but only *micronutrient supplements* stayed significant in the multivariate model (β=−0.03; SD = 0.01; *P* = 0.01), and their intake led to an 18% risk reduction in people within the highest compared with the lowest tertile of nutrient intake (RRR, 0.82; 95% CI, 0.57–1.20) (Table 3). We found no statistically significant association between taking micronutrients in supplemental form and progression of nuclear cataract.

## Discussion

This study found that progression of nuclear cataract over a 10-year period in a group of UK female twins is influenced by genetic factors that explain 35% of variance. The heritability estimate of cataract progression is lower than our previous cross-sectional estimates of susceptibility to development of nuclear cataract in this cohort,^29^ and it is also lower than the heritability estimated in the 324 individuals estimated from the nuclear score measurement at follow-up (61%; 95% CI, 45–72). This is consistent with previous studies showing that heritability generally is lower when examining change, compared with cross-sectional studies.^36^, ^37^, ^38^ In addition to early developmental differences and the body's response to environmental factors in adulthood, environmentally driven processes or accumulated “errors” (e.g., somatic gene mutation and epigenetic remodeling) might play a greater role in determining change during aging than genetic factors.^38^

This study also identified vitamin C as a micronutrient affecting nuclear cataract progression. We also replicate the previously found association between cross-sectional cataract and vitamin C intake. Vitamin C intake has long been studied in relation to age-related cataract because it is the L-enantiomer of ascorbate. A significant concentration of ascorbate is present in the aqueous humor that bathes the lens and may reduce oxidation products in the lens, thus reducing oxidative stress.^39^, ^40^ However, the conclusions of the many studies of the effects of ascorbate on cataract development are inconsistent and often conflicting.^6^, ^7^, ^8^, ^9^, ^10^, ^11^, ^12^, ^13^, ^14^, ^15^ Many of these have studied relatively well-nourished populations and are cross-sectional, although cross-sectional studies in India, where overall antioxidant levels may be lower, have found an inverse relationship between vitamin C and cataract.^9^, ^20^ Our results are similar to those of the Carotenoids in Age-Related Eye Disease Study that showed vitamin C intake, assessed with an FFQ 10 years before cataract assessment, to be protective of nuclear cataract prevalence.^15^ The Blue Mountains Eye Study also found that vitamin C intake, through both diet and supplements together, resulted in a lower nuclear cataract incidence over 10 years.^10^ This study is the first, to our knowledge, to show that dietary vitamin C intake protects against progression of nuclear lens opacity.

We also found dietary manganese to be protective against cross-sectional nuclear cataract independent of vitamin C. We cannot exclude that this association was a type I error, given we did not find an association between dietary manganese and nuclear cataract progression and the lack of a dose response (Table 3), although factors associated with incidence and progression do not always overlap. Manganese is an important antioxidant present in the human lens,^41^, ^42^, ^43^ and its concentration has been reported to be lower in cataractous lenses in comparison with normal lenses.^43^, ^44^ This study was not designed to elucidate the cause–effect relationship underlying the associations we found; therefore, we cannot distinguish whether manganese depletion is a cause or effect of cataractogenesis. Further studies are needed to answer this question. We also detected an association between supplemental intake of micronutrients and cross-sectional nuclear cataract but not between supplemental nutrients and cataract progression. These results are similar to those reported in the Blue Mountains Eye Study.^45^ Because only 10% or less of the participants in our study took any single supplement, we had to group supplements together; therefore, we could not draw conclusions on the effect of any single supplement or of components of supplements (e.g., supplemental vitamin C).

We used a highly quantitative measure of cataract from digital images (NDS), which essentially measures the nuclear opalescence (or “white scatter”) of the lens. The measure also was highly reproducible: the intraclass correlation coefficient for the worse eye in 30 subjects from our original study^29^ who came for repeat measurements was 0.93. The fact that every subject measured showed progression suggests that NDS is sensitive to change. Many epidemiologic studies have used the Lens Opacity Classification System (LOCS) grading scale, comparing phenotype with standardized photographs of 6 stages of lens opacification, which includes both nuclear opalescence and nuclear color or brunescence.^4^ The LOCS III was developed to increase steps between scores to allow greater sensitivity to change, accepting a lower intergrader reproducibility.^46^ Longitudinal studies using the LOCS III scale show relatively little change: In the Longitudinal Study of Cataract, only 24% of participants had an increase in nuclear opacities over an average of 4.6 years.^25^ Although our central NDS is not the same measure, it is highly correlated with average nuclear opalescence graded digitally or using a slit lamp.^29^ Digital image–derived NDSs using pixel density counts may be better suited for measuring progression and allowed our study the power to detect associations with a relatively small sample size.

### Study Limitations

A potential limitation is that our cohort is based on twin volunteers rather than a population study, but they are unselected and from across the United Kingdom and are unlikely to significantly differ from the UK general population.^47^ Twin studies use the “equal environment assumption” that the degree of shared family environment is the same for both MZ and DZ twin pairs. This is generally found to be true, although there are few studies of elderly subjects that explore this assumption. In addition, the TwinsUK cohort is predominantly a female cohort, and we could not assess any gender differences in risk factors. The findings of this study can be generalizable only to Caucasian women of similar age because they reflect cataract progression in a group of white British women between, on average, the ages of 60 and 70 years, and so may not reflect other population groups or age ranges. In this article, we explored the effect of all micronutrients on nuclear cataract formation; however, we had no data on carotenoid (lutein and zeaxanthin) intake. We also lacked power to explore the effects of smoking on cataract progression because 85% of participants have never smoked.

Those participants with follow-up data collected were seen as part of the HATS study, which was not designed as a cataract follow-up study. This meant that the number of subjects decreased to 324 individuals, thus reducing the amount of data we could analyze and our power. The individuals who were lost to follow-up in HATS were in general of lower socioeconomic status, had higher self-rated health status, and were less health aware.^31^ Any introduced bias probably would have resulted in loss of power because this group of individuals are more likely to have less healthy diets and more cataract. For this reason, we decided to test the association with progression only for nutrients that were associated with NDS at baseline. Those with follow-up data were on average 1.8 years younger than the original cohort, but in general they were not significantly different in other respects or in nutrient or supplement intake, hopefully reducing potential selection bias in the progression data. As in any observational study, ours is potentially susceptible to residual confounding, missing data, or misspecification of variables.

In summary, this study has shown that progression of nuclear cataract over a 10-year period is influenced by genetic factors with a heritability of 35%. Dietary vitamin C and manganese, both factors related to oxidative stress, seem to influence cross-sectional nuclear cataract, and vitamin C intake also significantly influences nuclear cataract progression.

## Figures and Tables

**Table 1:** Baseline Sample Characteristics and Nutrient Intakes in Individuals with or without Follow-up Data

	Subjects without Follow-up			Subjects with Follow-up		
Total	MZ	DZ	Total	MZ	DZ
No. of individuals	1730	827	916	324	151	173
Zygosity ratio (MZ:DZ)	1:1.1	-	-	1:1.2	-	-
Age (mean ± SD)	61.5±6.5	61.7±6.7	61.4±6.4	60.4±5.1	60.8±5.5	60.0±5.2
NDS (mean ± SD)	60.4±17.2	61.3±17.4	59.0±14.2	55.3±11.2	55.3±11.4	55.3±11.1
Sodium (mg)	2262.8±508.7	2265.3±476.3	2258.7±535.6	2237.4±456.4	2227.7±444.4	2247.2±444.4
Potassium (mg)	4013.5±637.4	3997.0±622.4	4026.9±650.6	4033.7±580.5	4094.5±588.4	3972.5±469.4
Calcium (mg)	1117.1±284.7	1118.5±284.9	1125.1±284.6	1118.9±291.5	1138.3±295.0	1099.4±568.0
Magnesium (mg)	347.3±56.4	347.3±56.8	347.2±56.0	343.8±55.0	347.0±58.0	340.6±287.5
Phosphorus (mg)	1527.1±247.0	1527.1±234.9	1527.1±257.8	1522.0±239.3	1532.0±251.0	1512.1±227.5
Iron (mg)∗	13.1±3.0	13.2±3.2	13.0±2.8	12.6±2.6	12.5±2.7	12.7±2.5
Copper (mg)	1.5±0.5	1.5±0.6	1.5±0.4	1.5±0.4	1.5±0.4	1.6±0.5
Zinc (mg)	10.2±1.7	10.2±1.8	10.1±1.7	10.2±1.7	10.2±1.8	10.1±1.6
Chloride (mg)	3629.6±792.9	3633.6±749.4	3623.0±828.6	3578.0±721.3	3566.7±690.3	3589.4±753.3
Manganese (mg)	4.2±1.2	4.1±1.1	4.2±1.2	4.2±1.1	4.3±1.1	4.2±1.1
Iodine (mg)	225.0±75.8	224.2±75.2	225.8±76.5	229.2±64.2	230.0±61.4	228.5±67.2
Retinol (μg)	579.5±817.8	569.1±570.6	554.8±496.6	611.8±472.9	588.2±422.6	635.6±519.0
Carotene (μg)	5343.4±3067.4	5503.7±3263.8	5200.4±2874.9	5305.6±3915.4	5663.8±4823.8	4945.0±2679.4
Vitamin D (μg)	2.7±1.4	2.7±1.1	2.6±1.5	2.8±1.1	3.0±1.0	2.6±1.0
Vitamin E (mg)	11.5±3.2	11.6±3.4	11.4±3.1	11.7±3.4	11.9±3.6	11.5±3.2
Thiamine (mg)∗	1.8±0.4	1.8±0.4	1.8±0.4	1.7±0.3	1.7±0.3	1.7±0.3
Riboflavin (mg)	2.5±0.7	2.4±0.7	2.5±0.7	2.4±0.6	2.5±0.6	2.4±0.7
Niacin (mg)	22.0±5.7	22.2±5.1	21.8±6.2	21.3±4.5	21.3±4.6	21.2±4.4
Tryptophan (mg)	17.4±3.0	17.5±2.7	17.3±3.3	17.2±2.5	17.3±2.5	17.1±2.6
Vitamin B6 (mg)	2.6±0.6	2.6±0.6	2.5±0.5	2.5±0.5	2.5±0.5	2.5±0.5
Vitamin B12 (μg)	6.5±3.2	6.7±3.6	6.4±2.9	6.7±2.3	6.7±2.3	6.7±2.4
Folate (μg)	402.2±113.1	400.7±114.0	403.2±112.3	395.7±98.9	402.0±95.9	389.4±101.8
Pantothenate (mg)	7.4±16.0	7.5±21.3	7.2±8.6	6.8±4.2	6.5±2.1	7.1±5.6
Biotin (mg)∗	48.1±10.5	47.7±10.3	48.5±10.8	49.7±10.3	50.6±10.2	48.7±10.3
Vitamin C (mg)	165.1±73.9	167.6±74.2	163.0±73.7	166.8±65.0	166.9±68.1	166.7±65.0
Any supplement (%)	55.1	54.8	55.4	55.0	54.1	55.9
Micronutrients (%)	32.57	32.4	33.2	31.7	32.8	30.8
Micronutrients excluding multivitamins (%)	23.6	24.1	23.2	21.6	24.2	19.3
Minerals only (%)	7.4	7.8	7.0	6.9	6.4	7.2
Other supplements (%)	44.9	46.2	44.4	47.1	44.2	49.5

DZ = dizygotic; MZ = monozygotic; NDS = nuclear dip score; SD = standard deviation.

The baseline characteristics of the participants and the baseline intake of micronutrients (mean ± standard deviation [SD]) and supplements per supplement group (% of users) are shown. The supplement groups studied are as follows: any supplement, micronutrient supplements (vitamins and mineral in any combination), micronutrient supplements excluding multivitamins (e.g., vitamin C only, vitamin D only, iron only, ACD complex), minerals only (e.g., iron only, calcium only), and other supplements (e.g., aloe vera, Echinacea, Ginkgo, omega-3).

∗ Denotes statistically significant difference (*P* < 0.05) between subjects with and without and without follow-up.

**Table 2:** Results from Univariable Regression Models

	Beta	Standard Error	*P* Value
	*Micronutrients*		
Sodium (mg)	5.41E-06	9.58E-06	0.56
Potassium (mg)∗	−1.58E-05	7.54E-06	0.04
Calcium (mg)	−1.95E-05	1.52E-05	0.20
Magnesium (mg)∗	−0.010	0.004	0.01
Phosphorus (mg)∗	−4.01E-05	1.94E-05	0.04
Iron (mg)	−1.15E-04	0.002	0.95
Copper (mg)	0.001	0.008	0.86
Zinc (mg)	−7.76E-04	0.003	0.77
Chloride (mg)	3.79E-06	6.10E-06	0.53
Manganese (mg)∗	−0.010	0.004	0.01
Iodine (mg)	−1.10E-04	6.07E-05	0.07
Retinol (μg)	2.36E-06	3.90E-06	0.55
Carotene (μg)	−1.67E-06	1.40E-06	0.23
Vitamin D (μg)	−0.004	0.003	0.22
Vitamin E (mg)∗	−0.003	0.001	0.04
Thiamine (mg)	−0.013	0.013	0.30
Riboflavin (mg)	−0.011	0.006	0.08
Niacin (mg)	−1.10E-04	8.26E-04	0.89
Tryptophan (mg)	−0.001	0.001	0.27
Vitamin B6 (mg)	−0.002	0.009	0.81
Vitamin B12 (μg)	−0.001	0.001	0.50
Folate (μg)∗	−9.91E-05	4.06E-05	0.02
Pantothenate (mg)	−2.81E-05	1.87E-04	0.88
Biotin (mg)	−3.01E-04	4.17E-04	0.47
Vitamin C (mg)∗	−1.742E-04	6.19E-05	0.01
	*Supplement Groups*†		
Any supplement	−0.015	0.009	0.12
Micronutrients∗	−0.032	0.013	0.01
Micronutrients excluding multivitamins	−0.023	0.012	0.06
Minerals only∗	−0.038	0.016	0.02
Any other supplement	0.005	0.014	0.72

The results of the univariable linear regression analysis between nuclear cataract (natural logarithm-transformed NDS) and energy-adjusted micronutrient intakes and between nuclear cataract and supplement intake per supplement group are shown.

∗ Denotes statistically significant associations at *P* < 0.05.

† Denotes that in the case of supplement groups, supplement intake was coded binary (presence vs. absence of intake of at least 1 of the components in the group). All analyses were adjusted for age and family structure.

**Table 3:** Results of Multinomial Regression Analysis for Factors Associated with Cross-Sectional Nuclear Cataract and Nuclear Cataract Progression

	**Cross-Sectional Results**			
	*Vitamin C*	*RRR*	*95% CI*	*P Value*
NDS Tertiles	34.5–53.2	reference		
53.3–54.5	0.89	0.77–1.02	0.09
54.6–229.2	0.81	0.68–0.96	0.01
	*Manganese*	*RRR*	*95% CI*	*P Value*
NDS Tertiles	34.5–53.2	reference		
53.3–54.5	0.76	0.66–0.87	0.001
54.6–229.2	0.8	0.67–0.95	0.01
	*Micronutrients*	*RRR*	*95% CI*	*P Value*
NDS Tertiles	34.5–53.2	reference		
53.3–54.5	0.82	0.60–1.12	0.82
54.6–229.2	0.82	0.57–1.20	0.82
	**Progression Results**			
	*Vitamin C*	*RRR*	*95% CI*	*P Value*
ΔNDS Tertiles	1.0–12.6	reference		
12.7–19.3	0.75	0.54–1.04	0.09
19.4–137.1	0.66	0.47–0.91	0.01

CI = confidence interval; NDS = nuclear dip score; ΔNDS = NDS at follow-up − NDS at baseline; RRR = relative risk ratio.

The results from the multinomial regression analysis for factors associated with cross-sectional (vitamin C and manganese) and progression (vitamin C) are shown. The RRR with its 95% CIs for each tertile of NDS or progression (ΔNDS) is reported. The minimum and maximum NDS score per tertile are reported.
